# 3,4,5-Trimeth­oxy­phenol

**DOI:** 10.1107/S1600536812042997

**Published:** 2012-10-20

**Authors:** Xiao-Chuan Jia, Jing Li, Zhi-Rui Yu, Hui Zhang, Lei Zhou

**Affiliations:** a Tianjin Entry–Exit Inspection and Quarantine Bureau, Tianjin 300201, People’s Republic of China

## Abstract

The asymmetric unit of the title compound, C_9_H_12_O_4_, consists of two crystallographically independent mol­ecules with similar conformations: essentially planar [r.m.s deviations for C_6_O_4_ = 0.0057 and 0.0137 Å] except for the central meth­oxy-methyl group [C—C—O—C torsion angles = 83.3 (2) and 83.9 (2)°]. In the crystal, O—H⋯O hydrogen bonds link the mol­ecules, generating supra­molecular chains along the *b* axis.The three-dimensional crystal structure is stabilized by C—H⋯O and C—H⋯π inter­actions.

## Related literature

For background information on the energetics and anti-oxidant potential of phenolic compounds, see: Matos *et al.* (2008 ▶); Gong *et al.* (2009 ▶).
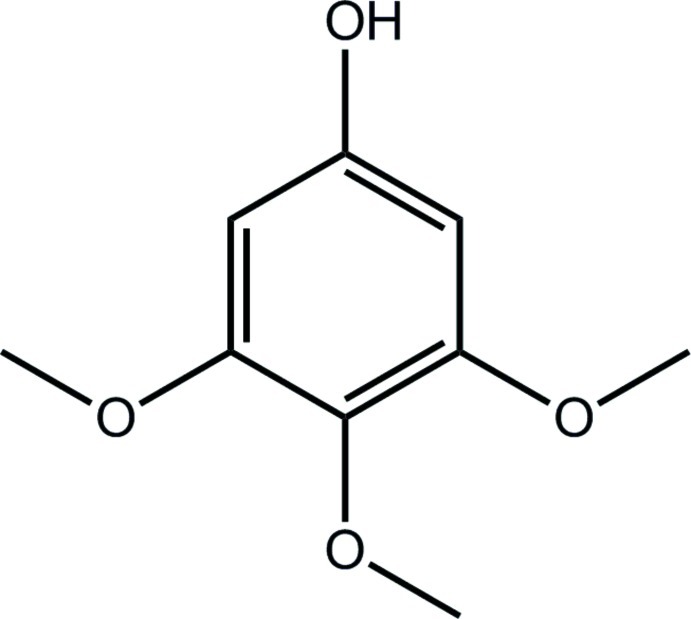


## Experimental

### 

#### Crystal data

C_9_H_12_O_4_
*M* *_r_* = 184.19
Monoclinic, 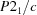

*a* = 15.355 (3) Å
*b* = 11.139 (2) Å
*c* = 11.546 (2) Å
β = 111.38 (3)°
*V* = 1839.0 (6) Å^3^
*Z* = 8
Mo *K*α radiation
μ = 0.11 mm^−1^
*T* = 296 K
0.20 × 0.15 × 0.10 mm

#### Data collection

Bruker SMART CCD area-detector diffractometer
Absorption correction: multi-scan (*SADABS*; Sheldrick, 1996 ▶) *T*_min_ = 0.981, *T*_max_ = 0.990
16747 measured reflections
3257 independent reflections
2957 reflections with *I* > 2σ(*I*)
*R*_int_ = 0.025

#### Refinement

*R*[*F* ^2^ > 2σ(*F* ^2^)] = 0.051
*wR*(*F* ^2^) = 0.123
*S* = 1.05
3257 reflections
243 parameters
H-atom parameters constrained
Δρ_max_ = 0.12 e Å^−3^
Δρ_min_ = −0.16 e Å^−3^

### 

Data collection: *SMART* (Bruker, 2007 ▶); cell refinement: *SAINT* (Bruker, 2007 ▶); data reduction: *SAINT*; program(s) used to solve structure: *SHELXS97* (Sheldrick, 2008 ▶); program(s) used to refine structure: *SHELXL97* (Sheldrick, 2008 ▶); molecular graphics: *SHELXTL* (Sheldrick, 2008 ▶); software used to prepare material for publication: *SHELXTL*.

## Supplementary Material

Crystal structure: contains datablock(s) I, global. DOI: 10.1107/S1600536812042997/tk5160sup1.cif

Structure factors: contains datablock(s) I. DOI: 10.1107/S1600536812042997/tk5160Isup2.hkl

Supplementary material file. DOI: 10.1107/S1600536812042997/tk5160Isup3.cml

Additional supplementary materials:  crystallographic information; 3D view; checkCIF report

## Figures and Tables

**Table 1:** Hydrogen-bond geometry (Å, °) *Cg*1 and *Cg*2 are the centroids of the C1–C3,C5,C7,C9 and C10–C12,C14,C16,C18 benzene rings, respectively.

*D*—H⋯*A*	*D*—H	H⋯*A*	*D*⋯*A*	*D*—H⋯*A*
O5—H5⋯O7^i^	0.82	1.93	2.7484 (18)	179
O1—H1⋯O3^ii^	0.82	1.90	2.7204 (17)	175
C6—H6*A*⋯O1^iii^	0.96	2.57	3.256 (3)	129
C15—H15*A*⋯O5^iv^	0.96	2.59	3.270 (3)	128
C4—H4*B*⋯*Cg*1^v^	0.96	2.86	3.777 (2)	160
C17—H17*B*⋯*Cg*2^vi^	0.96	2.85	3.736 (2)	154
C13—H13*B*⋯O1^vii^	0.96	2.49	3.303 (3)	142

Symmetry codes: (i) 
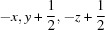
; (ii) 
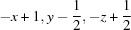
; (iii) 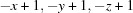; (iv) 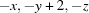; (v) 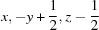; (vi) 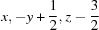; (vii) 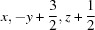.

**Fig. 1.**
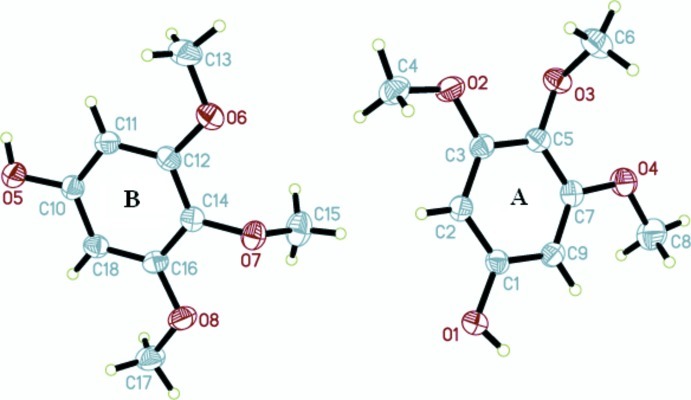
The molecular structure of the title compound, with displacement ellipsoids at the 30% probability level.

**Fig. 2.**
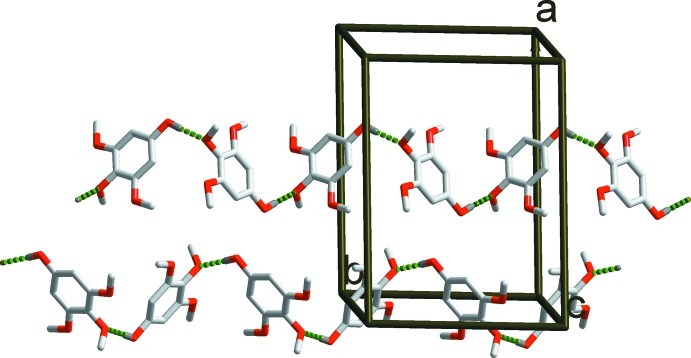
A potion of the unit cell contents highlighting the chain structure of the title compound, linked via O—H···O hydrogen bonds (dashed lines). H atoms have been omitted for clarity, except for those involved in hydrogen-bonded interactions.

## supplementary crystallographic information

### Comment

The study of the energetics of phenolic compounds (Matos *et al.*, 2008) has considerable practical interest since this class of chemical compound includes a large number of synthetic and naturally occurring antioxidants. They inhibit the oxidation of materials of both commercial and biological importance. This antioxidant function is due to the ability of phenols to trap the peroxyl radicals *via* the hydrogen transfer reaction (Gong *et al.*, 2009). In order to expand this field, we now report the structure of the title compound.

The molecule of the title compound (Fig. 1), consists of two crystallographically independent molecules, A and B, with similar conformations. All O-atoms in both molecules are coplanar with the benzene rings they are attached to, and the mean r.s.m in molecules A and B are 0.0057 and 0.0137 Å, respectively.

In the crystal, it is worth mentioning that strong intermolecular O—H···O hydrogen bonds link molecules A and B to generate a one dimensional chain (Fig. 2 and Table 1) along the *b* axis. These are connected into a supramolecular layer in the *bc* plane by C—H···O and C-H···π interactions (Table 1). The layers are connected into a three-dimensional crystal structure by C—H···O hydrogen bonds (Table 2) involving the C13 and O1 atoms (Table 1).

### Experimental

3,4,5-Trimethoxyphenol was obtained commercially from Aldrich Chemical Co. Single crystals suitable for X-ray diffraction were obtained by recrystallizing the prude product from its chloroform solution by slow evaporation at room temperature over a period of seven days.

### Refinement

All H atoms were placed in idealized positions (C—H = 0.93–0.96 Å, O—H = 0.82 Å) and refined as riding atoms with *U*_iso_(H) = 1.2*U*_eq_(C) and *U*_iso_(H) = 1.5*U*_eq_(O).

### Crystal data

**Table:**

C_9_H_12_O_4_	*F*(000) = 784
*M**_r_* = 184.19	*D*_x_ = 1.331 Mg m^−^^3^
Monoclinic, *P*2_1_/*c*	Mo *K*α radiation, λ = 0.71073 Å
Hall symbol: -P 2ybc	Cell parameters from 5944 reflections
*a* = 15.355 (3) Å	θ = 3.3–25.4°
*b* = 11.139 (2) Å	µ = 0.11 mm^−^^1^
*c* = 11.546 (2) Å	*T* = 296 K
β = 111.38 (3)°	Block, colourless
*V* = 1839.0 (6) Å^3^	0.20 × 0.15 × 0.10 mm
*Z* = 8	

### Data collection

**Table:**

Bruker SMART CCD area-detector diffractometer	3257 independent reflections
Radiation source: fine-focus sealed tube	2957 reflections with *I* > 2σ(*I*)
Graphite monochromator	*R*_int_ = 0.025
φ and ω scans	θ_max_ = 25.1°, θ_min_ = 3.3°
Absorption correction: multi-scan (*SADABS*; Sheldrick, 1996)	*h* = −18→17
*T*_min_ = 0.981, *T*_max_ = 0.990	*k* = −13→13
16747 measured reflections	*l* = −11→13

### Refinement

**Table:**

Refinement on *F*^2^	Primary atom site location: structure-invariant direct methods
Least-squares matrix: full	Secondary atom site location: difference Fourier map
*R*[*F*^2^ > 2σ(*F*^2^)] = 0.051	Hydrogen site location: inferred from neighbouring sites
*wR*(*F*^2^) = 0.123	H-atom parameters constrained
*S* = 1.05	*w* = 1/[σ^2^(*F*_o_^2^) + (0.0545*P*)^2^ + 0.3972*P*] where *P* = (*F*_o_^2^ + 2*F*_c_^2^)/3
3257 reflections	(Δ/σ)_max_ < 0.001
243 parameters	Δρ_max_ = 0.12 e Å^−^^3^
0 restraints	Δρ_min_ = −0.16 e Å^−^^3^

### Special details

**Table:**

Geometry. All esds (except the esd in the dihedral angle between two l.s. planes) are estimated using the full covariance matrix. The cell esds are taken into account individually in the estimation of esds in distances, angles and torsion angles; correlations between esds in cell parameters are only used when they are defined by crystal symmetry. An approximate (isotropic) treatment of cell esds is used for estimating esds involving l.s. planes.
Refinement. Refinement of F^2^ against ALL reflections. The weighted R-factor wR and goodness of fit S are based on F^2^, conventional R-factors R are based on F, with F set to zero for negative F^2^. The threshold expression of F^2^ > 2sigma(F^2^) is used only for calculating R-factors(gt) etc. and is not relevant to the choice of reflections for refinement. R-factors based on F^2^ are statistically about twice as large as those based on F, and R- factors based on ALL data will be even larger.

### Fractional atomic coordinates and isotropic or equivalent isotropic displacement parameters (Å^2^)

**Table:**

	*x*	*y*	*z*	*U*_iso_*/*U*_eq_	
O1	0.33376 (8)	0.41617 (11)	0.19861 (12)	0.0525 (3)	
H1	0.3521	0.3629	0.1642	0.079*	
O4	0.64034 (9)	0.58079 (12)	0.27042 (13)	0.0602 (4)	
O3	0.61501 (8)	0.74092 (10)	0.42789 (11)	0.0509 (3)	
C2	0.39182 (12)	0.57694 (14)	0.33613 (15)	0.0445 (4)	
H2	0.3366	0.5772	0.3520	0.053*	
C3	0.46239 (12)	0.65836 (14)	0.39471 (15)	0.0443 (4)	
C5	0.54480 (11)	0.65824 (14)	0.37034 (15)	0.0443 (4)	
C7	0.55643 (11)	0.57554 (15)	0.28722 (15)	0.0447 (4)	
O2	0.45748 (9)	0.74281 (11)	0.47812 (13)	0.0579 (4)	
C9	0.48632 (11)	0.49346 (15)	0.22769 (15)	0.0452 (4)	
H9	0.4938	0.4384	0.1715	0.054*	
C1	0.40488 (11)	0.49505 (14)	0.25354 (15)	0.0426 (4)	
C8	0.64850 (16)	0.5093 (2)	0.1731 (2)	0.0805 (7)	
H8A	0.6423	0.4261	0.1903	0.121*	
H8B	0.7085	0.5225	0.1673	0.121*	
H8C	0.6002	0.5309	0.0958	0.121*	
C6	0.67555 (14)	0.7061 (2)	0.55005 (19)	0.0676 (6)	
H6A	0.6420	0.7098	0.6057	0.101*	
H6B	0.7281	0.7596	0.5786	0.101*	
H6C	0.6972	0.6256	0.5478	0.101*	
C4	0.38077 (15)	0.73582 (19)	0.5198 (2)	0.0644 (5)	
H4A	0.3235	0.7519	0.4514	0.097*	
H4B	0.3890	0.7940	0.5843	0.097*	
H4C	0.3782	0.6568	0.5517	0.097*	
O7	0.11271 (9)	0.75191 (10)	0.18255 (12)	0.0519 (3)	
O5	−0.16694 (8)	1.08198 (11)	0.13218 (12)	0.0557 (3)	
H5	−0.1501	1.1323	0.1878	0.084*	
O6	0.13740 (8)	0.91453 (12)	0.36143 (12)	0.0587 (4)	
C16	−0.03779 (12)	0.83857 (15)	0.06267 (15)	0.0462 (4)	
C12	0.05455 (11)	0.92090 (15)	0.26236 (15)	0.0440 (4)	
C18	−0.10754 (12)	0.92213 (15)	0.05119 (16)	0.0479 (4)	
H18	−0.1617	0.9235	−0.0197	0.058*	
C14	0.04348 (11)	0.83728 (14)	0.16863 (15)	0.0439 (4)	
C11	−0.01507 (11)	1.00391 (15)	0.25244 (15)	0.0456 (4)	
H11	−0.0079	1.0590	0.3158	0.055*	
O8	−0.04248 (9)	0.75347 (11)	−0.02475 (12)	0.0606 (4)	
C10	−0.09552 (11)	1.00336 (15)	0.14650 (16)	0.0450 (4)	
C17	−0.11899 (15)	0.75973 (19)	−0.14040 (19)	0.0659 (6)	
H17A	−0.1765	0.7496	−0.1266	0.099*	
H17B	−0.1131	0.6974	−0.1945	0.099*	
H17C	−0.1190	0.8365	−0.1782	0.099*	
C15	0.17645 (16)	0.7822 (2)	0.1232 (2)	0.0735 (6)	
H15A	0.1450	0.7779	0.0346	0.110*	
H15B	0.2279	0.7268	0.1487	0.110*	
H15C	0.1994	0.8622	0.1463	0.110*	
C13	0.14821 (16)	0.9927 (2)	0.46273 (19)	0.0749 (6)	
H13A	0.1435	1.0744	0.4348	0.112*	
H13B	0.2084	0.9798	0.5267	0.112*	
H13C	0.1001	0.9766	0.4953	0.112*	

### Atomic displacement parameters (Å^2^)

**Table:**

	*U*^11^	*U*^22^	*U*^33^	*U*^12^	*U*^13^	*U*^23^
O1	0.0433 (6)	0.0543 (8)	0.0560 (7)	−0.0037 (5)	0.0135 (6)	−0.0136 (6)
O4	0.0501 (7)	0.0696 (9)	0.0688 (9)	−0.0055 (6)	0.0311 (7)	−0.0059 (7)
O3	0.0527 (7)	0.0453 (7)	0.0509 (7)	−0.0101 (5)	0.0143 (6)	0.0044 (5)
C2	0.0429 (9)	0.0426 (9)	0.0496 (10)	0.0027 (7)	0.0186 (8)	0.0017 (7)
C3	0.0492 (9)	0.0375 (9)	0.0460 (9)	0.0017 (7)	0.0172 (7)	0.0010 (7)
C5	0.0463 (9)	0.0387 (9)	0.0454 (9)	−0.0035 (7)	0.0139 (7)	0.0049 (7)
C7	0.0438 (9)	0.0461 (10)	0.0458 (9)	0.0033 (7)	0.0182 (7)	0.0074 (7)
O2	0.0614 (8)	0.0503 (7)	0.0698 (9)	−0.0090 (6)	0.0330 (7)	−0.0185 (6)
C9	0.0490 (9)	0.0439 (9)	0.0427 (9)	0.0047 (8)	0.0167 (7)	−0.0002 (7)
C1	0.0406 (8)	0.0418 (9)	0.0408 (8)	0.0029 (7)	0.0092 (7)	0.0035 (7)
C8	0.0706 (14)	0.0924 (17)	0.0975 (17)	−0.0050 (12)	0.0533 (13)	−0.0198 (14)
C6	0.0596 (12)	0.0693 (13)	0.0594 (12)	−0.0081 (10)	0.0047 (10)	0.0066 (10)
C4	0.0720 (13)	0.0632 (12)	0.0698 (13)	−0.0076 (10)	0.0399 (11)	−0.0177 (10)
O7	0.0555 (7)	0.0461 (7)	0.0579 (8)	0.0084 (5)	0.0253 (6)	0.0097 (5)
O5	0.0506 (7)	0.0549 (8)	0.0590 (8)	0.0072 (6)	0.0167 (6)	−0.0080 (6)
O6	0.0483 (7)	0.0689 (9)	0.0505 (7)	0.0006 (6)	0.0079 (6)	−0.0046 (6)
C16	0.0547 (10)	0.0395 (9)	0.0449 (9)	−0.0035 (8)	0.0188 (8)	−0.0016 (7)
C12	0.0416 (9)	0.0468 (10)	0.0434 (9)	−0.0064 (7)	0.0151 (7)	0.0038 (7)
C18	0.0481 (9)	0.0477 (10)	0.0437 (9)	−0.0014 (8)	0.0117 (8)	−0.0004 (7)
C14	0.0459 (9)	0.0402 (9)	0.0478 (9)	0.0005 (7)	0.0197 (8)	0.0048 (7)
C11	0.0493 (10)	0.0442 (9)	0.0448 (9)	−0.0058 (8)	0.0189 (8)	−0.0047 (7)
O8	0.0676 (8)	0.0514 (8)	0.0547 (8)	0.0062 (6)	0.0126 (6)	−0.0130 (6)
C10	0.0444 (9)	0.0419 (9)	0.0516 (10)	−0.0010 (7)	0.0210 (8)	0.0019 (7)
C17	0.0733 (13)	0.0658 (13)	0.0511 (11)	0.0015 (10)	0.0139 (10)	−0.0141 (9)
C15	0.0728 (14)	0.0749 (14)	0.0894 (16)	0.0121 (11)	0.0492 (13)	0.0126 (12)
C13	0.0688 (13)	0.0847 (16)	0.0551 (12)	−0.0053 (12)	0.0034 (10)	−0.0148 (11)

### Geometric parameters (Å, º)

**Table:**

O1—C1	1.364 (2)	O7—C14	1.391 (2)
O1—H1	0.8200	O7—C15	1.425 (2)
O4—C7	1.372 (2)	O5—C10	1.366 (2)
O4—C8	1.420 (2)	O5—H5	0.8200
O3—C5	1.389 (2)	O6—C12	1.368 (2)
O3—C6	1.431 (2)	O6—C13	1.418 (2)
C2—C3	1.386 (2)	C16—O8	1.367 (2)
C2—C1	1.386 (2)	C16—C18	1.388 (2)
C2—H2	0.9300	C16—C14	1.393 (2)
C3—O2	1.368 (2)	C12—C11	1.386 (2)
C3—C5	1.393 (2)	C12—C14	1.391 (2)
C5—C7	1.388 (2)	C18—C10	1.384 (2)
C7—C9	1.389 (2)	C18—H18	0.9300
O2—C4	1.428 (2)	C11—C10	1.386 (2)
C9—C1	1.388 (2)	C11—H11	0.9300
C9—H9	0.9300	O8—C17	1.423 (2)
C8—H8A	0.9600	C17—H17A	0.9600
C8—H8B	0.9600	C17—H17B	0.9600
C8—H8C	0.9600	C17—H17C	0.9600
C6—H6A	0.9600	C15—H15A	0.9600
C6—H6B	0.9600	C15—H15B	0.9600
C6—H6C	0.9600	C15—H15C	0.9600
C4—H4A	0.9600	C13—H13A	0.9600
C4—H4B	0.9600	C13—H13B	0.9600
C4—H4C	0.9600	C13—H13C	0.9600
			
C1—O1—H1	109.5	C14—O7—C15	114.37 (14)
C7—O4—C8	116.51 (15)	C10—O5—H5	109.5
C5—O3—C6	113.81 (13)	C12—O6—C13	116.83 (15)
C3—C2—C1	118.89 (16)	O8—C16—C18	124.27 (16)
C3—C2—H2	120.6	O8—C16—C14	115.49 (15)
C1—C2—H2	120.6	C18—C16—C14	120.24 (15)
O2—C3—C2	124.04 (15)	O6—C12—C11	123.91 (15)
O2—C3—C5	115.48 (15)	O6—C12—C14	115.38 (15)
C2—C3—C5	120.48 (15)	C11—C12—C14	120.71 (15)
C7—C5—O3	119.84 (15)	C10—C18—C16	119.19 (16)
C7—C5—C3	119.71 (15)	C10—C18—H18	120.4
O3—C5—C3	120.44 (15)	C16—C18—H18	120.4
O4—C7—C5	115.70 (15)	C12—C14—O7	119.76 (15)
O4—C7—C9	123.82 (15)	C12—C14—C16	119.52 (15)
C5—C7—C9	120.48 (15)	O7—C14—C16	120.71 (15)
C3—O2—C4	117.63 (14)	C10—C11—C12	118.84 (15)
C1—C9—C7	118.82 (15)	C10—C11—H11	120.6
C1—C9—H9	120.6	C12—C11—H11	120.6
C7—C9—H9	120.6	C16—O8—C17	117.52 (14)
O1—C1—C2	116.85 (15)	O5—C10—C18	117.03 (15)
O1—C1—C9	121.55 (15)	O5—C10—C11	121.48 (15)
C2—C1—C9	121.61 (15)	C18—C10—C11	121.49 (16)
O4—C8—H8A	109.5	O8—C17—H17A	109.5
O4—C8—H8B	109.5	O8—C17—H17B	109.5
H8A—C8—H8B	109.5	H17A—C17—H17B	109.5
O4—C8—H8C	109.5	O8—C17—H17C	109.5
H8A—C8—H8C	109.5	H17A—C17—H17C	109.5
H8B—C8—H8C	109.5	H17B—C17—H17C	109.5
O3—C6—H6A	109.5	O7—C15—H15A	109.5
O3—C6—H6B	109.5	O7—C15—H15B	109.5
H6A—C6—H6B	109.5	H15A—C15—H15B	109.5
O3—C6—H6C	109.5	O7—C15—H15C	109.5
H6A—C6—H6C	109.5	H15A—C15—H15C	109.5
H6B—C6—H6C	109.5	H15B—C15—H15C	109.5
O2—C4—H4A	109.5	O6—C13—H13A	109.5
O2—C4—H4B	109.5	O6—C13—H13B	109.5
H4A—C4—H4B	109.5	H13A—C13—H13B	109.5
O2—C4—H4C	109.5	O6—C13—H13C	109.5
H4A—C4—H4C	109.5	H13A—C13—H13C	109.5
H4B—C4—H4C	109.5	H13B—C13—H13C	109.5
			
C1—C2—C3—O2	−179.75 (15)	C13—O6—C12—C11	4.7 (2)
C1—C2—C3—C5	0.4 (2)	C13—O6—C12—C14	−175.54 (17)
C6—O3—C5—C7	−97.21 (19)	O8—C16—C18—C10	179.30 (16)
C6—O3—C5—C3	83.3 (2)	C14—C16—C18—C10	−0.4 (3)
O2—C3—C5—C7	179.80 (15)	O6—C12—C14—O7	2.6 (2)
C2—C3—C5—C7	−0.3 (2)	C11—C12—C14—O7	−177.60 (14)
O2—C3—C5—O3	−0.7 (2)	O6—C12—C14—C16	−178.57 (15)
C2—C3—C5—O3	179.16 (14)	C11—C12—C14—C16	1.2 (2)
C8—O4—C7—C5	−171.18 (17)	C15—O7—C14—C12	−97.3 (2)
C8—O4—C7—C9	9.5 (3)	C15—O7—C14—C16	83.9 (2)
O3—C5—C7—O4	1.5 (2)	O8—C16—C14—C12	179.76 (15)
C3—C5—C7—O4	−179.02 (15)	C18—C16—C14—C12	−0.5 (2)
O3—C5—C7—C9	−179.10 (14)	O8—C16—C14—O7	−1.5 (2)
C3—C5—C7—C9	0.4 (2)	C18—C16—C14—O7	178.29 (15)
C2—C3—O2—C4	8.9 (2)	O6—C12—C11—C10	178.80 (15)
C5—C3—O2—C4	−171.24 (16)	C14—C12—C11—C10	−0.9 (2)
O4—C7—C9—C1	178.85 (15)	C18—C16—O8—C17	7.3 (3)
C5—C7—C9—C1	−0.5 (2)	C14—C16—O8—C17	−173.01 (16)
C3—C2—C1—O1	179.64 (14)	C16—C18—C10—O5	−178.55 (15)
C3—C2—C1—C9	−0.5 (2)	C16—C18—C10—C11	0.7 (3)
C7—C9—C1—O1	−179.58 (14)	C12—C11—C10—O5	179.19 (15)
C7—C9—C1—C2	0.6 (2)	C12—C11—C10—C18	0.0 (2)

### Hydrogen-bond geometry (Å, º)

Cg1 and Cg2 are the centroids of the C1–C3,C5,C7,C9 and C10–C12,C14,C16,C18 benzene rings, respectively.

**Table:**

*D*—H···*A*	*D*—H	H···*A*	*D*···*A*	*D*—H···*A*
O5—H5···O7^i^	0.82	1.93	2.7484 (18)	179
O1—H1···O3^ii^	0.82	1.90	2.7204 (17)	175
C6—H6*A*···O1^iii^	0.96	2.57	3.256 (3)	129
C15—H15*A*···O5^iv^	0.96	2.59	3.270 (3)	128
C4—H4*B*···*Cg*1^v^	0.96	2.86	3.777 (2)	160
C17—H17*B*···*Cg*2^vi^	0.96	2.85	3.736 (2)	154
C13—H13*B*···O1^vii^	0.96	2.49	3.303 (3)	142

Symmetry codes: (i) −*x*, *y*+1/2, −*z*+1/2; (ii) −*x*+1, *y*−1/2, −*z*+1/2; (iii) −*x*+1, −*y*+1, −*z*+1; (iv) −*x*, −*y*+2, −*z*; (v) *x*, −*y*+1/2, *z*−1/2; (vi) *x*, −*y*+1/2, *z*−3/2; (vii) *x*, −*y*+3/2, *z*+1/2.
